# Determinants of blood sugar level among type I diabetic patients in Debre Tabor General Hospital, Ethiopia: a longitudinal study

**DOI:** 10.1038/s41598-022-12891-1

**Published:** 2022-05-31

**Authors:** Molalign Gualu Gobena, Maru Zewdu Kassie

**Affiliations:** Department of Statistics, Natural and Computational Sciences, Assosa University, Asosa, Ethiopia

**Keywords:** Diseases, Medical research, Risk factors

## Abstract

In 2019 among all populous countries in Africa, Ethiopia has the fourth-highest number of people with diabetes (1.7 million). This aggravated prevalence figure implies that diabetes mellitus is a major public health problem in Ethiopia. Due to urbanization, this problem is very critical in the Amhara region, Ethiopia. The study aimed to identify factors that affect the longitudinal fasting blood sugar among T1DM (Type I diabetes mellitus) patients in Debre Tabor General Hospital (DTGH); North-west Ethiopia. A retrospective study design was conducted from 210 randomly selected T1DM patients in the clinic (Outpatient Department) at Debre Tabor General Hospital under the follow-up period from September 2019 to August 2021. To fit these retrospective data records, we used Random intercept and slope models. In this study, the unstructured variance–covariance structure was the appropriate structure for the random intercept and slope model. At a 5% level of significance, family history of diabetes mellitus, age, comorbidity, hemoglobin, and visit time in months were significant factors. Also, all the random effect parameters were statistically significant. It implies that the variability within and between T1DM patients in FBS over time was statistically significant. The mean fasting blood sugar level at baseline was 5.4944 mg/dl and decreased to 5.0679 mg/dl at the final follow-up time. Major contributors for the increment of fasting blood sugar level were increasing age, decreasing haemoglobin, having comorbidity, and belonging from a family with diabetes history. The overall within and between variability in fasting blood sugar level among T1DM patients in DTGH were high. Intervention measures at DTGH level should be undertaken using health education and other measures by providing an emphasis on the prevention, early detection, and treatment of diabetes mellitus.

## Introduction

Diabetes mellitus is a condition in which the body fails to generate or respond to insulin effectively. It is a major public health problem responsible for morbidity and mortality^1^. It orders the patient to be economically dependent on their family^2^. Type 1 diabetes also increased rapidly with many complications^2,3^. This problem is also critical in Ethiopia especially in the study area^4^. It is also a lifetime challenge that requires proper self-care practices for better glycemic control. Insulin injection, dietary restriction, and physical exercises are some of the interventions intended to slow down the progress of diabetes mellitus^5^.

The epidemiological dynamics of diabetes have changed dramatically day today. In 2019 the global prevalence was estimated to be 9.3% (463 million people). This quantity will rise to 10.2% (578 million) and 10.9% (700 million) by 2030 and 2045 respectively. In a developed country, the prevalence will rise (10.4%) more than the developing countries (4.0%)^6^. Also almost half of the people living with diabetes do not know that they have diabetes^6^. In many African countries, the number of cases and number of death are low compared to European and American countries; this could be due to underreporting and low test capacity. However, on the ground now it is a common problem in Africa specifically in Ethiopia^7^. In 2019 among all populous countries in Africa, Ethiopia has the fourth-highest number of people with diabetes (1.7 million). For instance, the number of people with diabetes (in million) in South Africa, Nigeria, and the Democratic Republic of Congo are 4.6, 2.7, and 1.8 respectively^8^. This aggravated prevalence figure in Ethiopia implies that diabetes mellitus is a major public health problem in Ethiopia^7,8^.

Knowing the T1DM disease progression helps us to understand the severity of the disease and to identify the determinant factors for its glycaemic control. The previous studies showed that among T1DM patients with a similar level of glycemia, iron deficiency anemia was associated with higher concentrations of HbA1c^9–12^. In chronic cases like diabetes mellitus, hypertension, stroke, and other related diseases the repeatedly measured variables are important to identify the disease progression, specifically, in diabetes mellitus, the blood sugar levels of the patients are measured during the follow-up time approximately in 3 months interval. Their sugar level may increase or decrease depending on the patient's self-care management, to analyze these continuous data the linear mixed model is the best way^13^. Therefore, this study focused on identifying factors that affect the longitudinal fasting blood sugar of T1DM patients attending the OPD section at DTGH using linear mixed model analysis.

## Methods

In this study, all methods were performed in accordance with the relevant guidelines and regulations.

### Data description and study design

A retrospective study design was carried out from September 2019–August 2021 in DTGH to conduct this study. Relevant information was extracted from the medical records of T1DM patients. The study population for this study was T1DM patients who were under the follow up of insulin medication at DTGH who start medication in September 2019 and followed up to August 2021. Here there were 604 T1DM patients in the study period. From these, only 210 patients were with relevant information. These 210 patients were followed until they got either the event or censored.

The response variable in the current investigation was fasting blood sugar level measured in mg/dl. The longitudinal outcome variable FBS (Fasting Blood Sugar) is measured approximately every 3 months irrespective of patient visits to the OPD section of chronic disease at DTGH. that is; at the start of the treatment, 0-, 3-, 6-, 9-, 12-, 15-, 18-, 21- and 24-month visits (that means n_i_ = 9). The sample sizes at these 9-time points are (210, 210, 210, 210, 195, 178, 151, 116, 57) which show up to third follow up the sample sizes were constant but after the third follow up it shows a high degree of missing data over time due to patients attained the first recovery. Age, Gender, Place of residence, Presence of comorbidity, Family history of DM, Creatinine, Weight, Visit time and Haemoglobin were the potential explanatory considered in this study (Table 1).

**Table 1:** The mode, scale and nature of the variables in this study.

S/N	Variable	Description
1	Age	Year
2	Gender	Female = 0, male = 1
3	Place of residence	Rural = 0, urban = 1
4	Comorbidity	No = 0, yes = 1
5	Family history of DM	No = 0, yes = 1
6	Hemoglobin	Measured in g/dL
7	Creatinine	Measured in mg/dL
8	Visit time	Month (0, 3, 6, 9, 12, 15, 18, 21, 24)
9	Blood sugar level	Measured in mg/dl

### Longitudinal data analysis

**Figure1:**
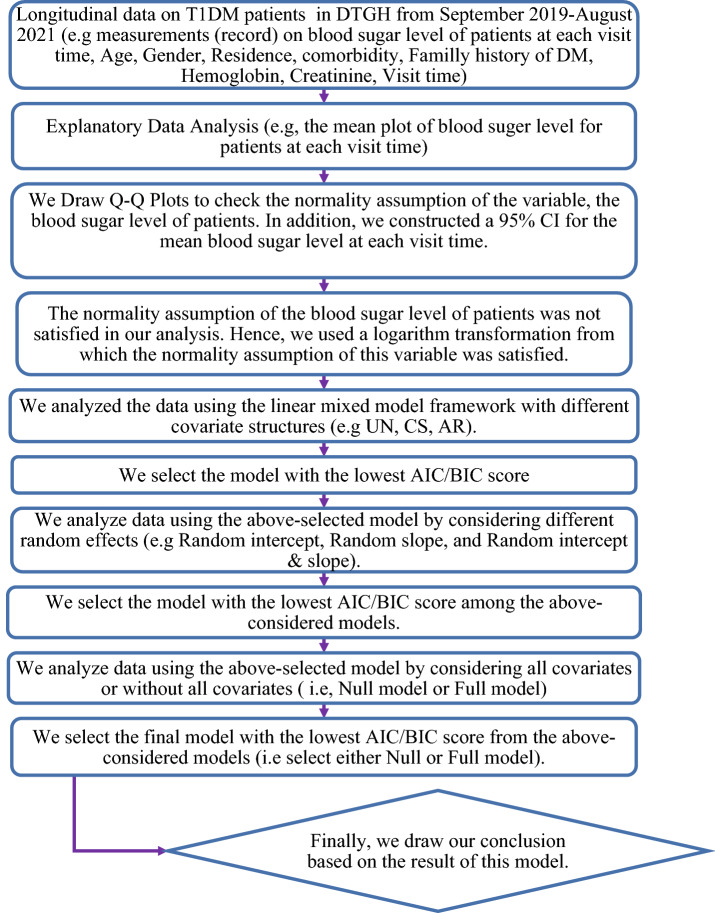
Flowchart of longitudinal data analysis in this retrospective study.

Figure 1 shows a flowchart of longitudinal data analysis in this retrospective study. The flow chart included 11 stages, and these stages described as follows. *Stage 1* A longitudinal data on T1DM patients in DTGH were collected based on our variable of interest in this study. *Stage 2* We have drawn the mean plot of fasting blood sugar level for T1DM patients at each follow up time (visit time). This plot helps to know the variability of fasting blood sugar level among T1DM patients in DTGH over visit time. In addition, this is also helpful to observe the mean of fasting blood sugar level for T1DM patients in DTGH over visit time, and to check the linearity assumption of the variable (fasting blood sugar level)^14,15^. *Stage 3* We used Q-Q plot to check the normality assumption of the variable (fasting blood sugar level). It is one of the requirements of the linear mixed model. We also constructed a 95% CI for the mean blood sugar level at each visit time. It is helpful to estimate the interval which holds the mean blood sugar level of TIDM patients in DTGH^14,15^. *Stage 4* We used the logarithm transformation to fulfill the normality assumption of the variable (fasting blood sugar level)^14^. *Stage 5* At this stage, different covariate structure (e.g. UN, CS, AR) was considered to analysis the data using linear mixed model framework. This procedure helps to fit model with more parsimonious structures by selecting appropriate variance–covariance structure of Σ^16^. *Stage 6* At this stage, we select model with appropriate variance–covariance structure of Σ^16,17^. *Stage 7* At this stage, we analyze data using the selected model in stage 6 by considering different random effects. This procedure helps to provide differed random effect models (e.g. Random intercept, Random slope, and Random intercept & slope)^14,15,17^. *Stage 8* We have selected one of the above suggested model in stage 7 using AIC/BIC criteria^17^. *Stage 9* We analyze the data using the two version of the above selected model in stage 8 (Null model or Full model)^14,15,17^. *Stage 10* We have selected the final model from the two version considered in stage 9^17^. *Stage 11* Finally, we draw our conclusion based on the result of this final model (Fig. 1).

### Weakness of the linear mixed model

Even though the model has several advantages in longitudinal data analysis, it has amiss. First, more assumptions on distribution should be made and then, approximations are used to estimate the parameter of this model. This implies that the result is highly dependent on more distributional assumptions. Consequently, if the assumptions entirely not satisfied, it may lead to biased parameter estimates^18^.

### Ethics approval and consent to participate

We have got a permission letter with Ref ≠ 2019-RCS-006 from the Ethical approval committee of statistics department in Assosa University, Ethiopia to conduct this study by use of this secondary data. When the data collected primarily, subjects were properly instructed, and they provided informed consent to participate by signing the appropriate consent form. It was assured by the Ethical approval committee and DTGH Managers.

## Results

The data was analyzed using R-4.00 and SAS Version 9.2.

### Longitudinal data exploration

**Figure 2:**
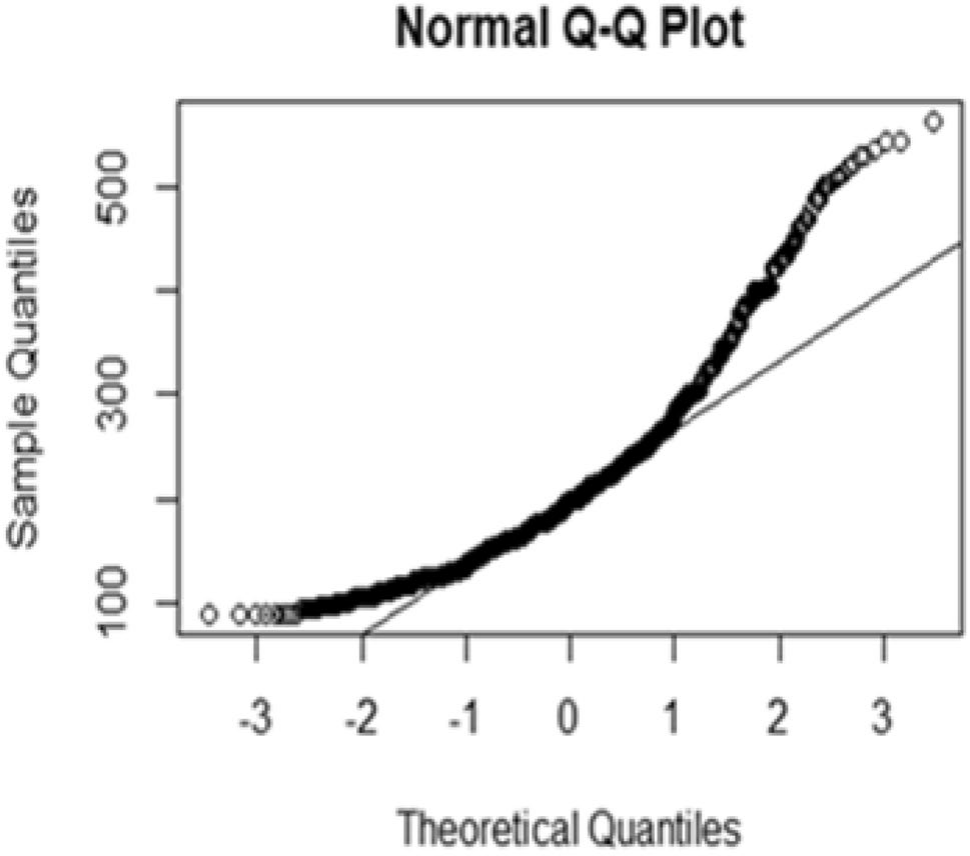
Q–Q plot of the actual FBS measurements over time.

**Figure 3:**
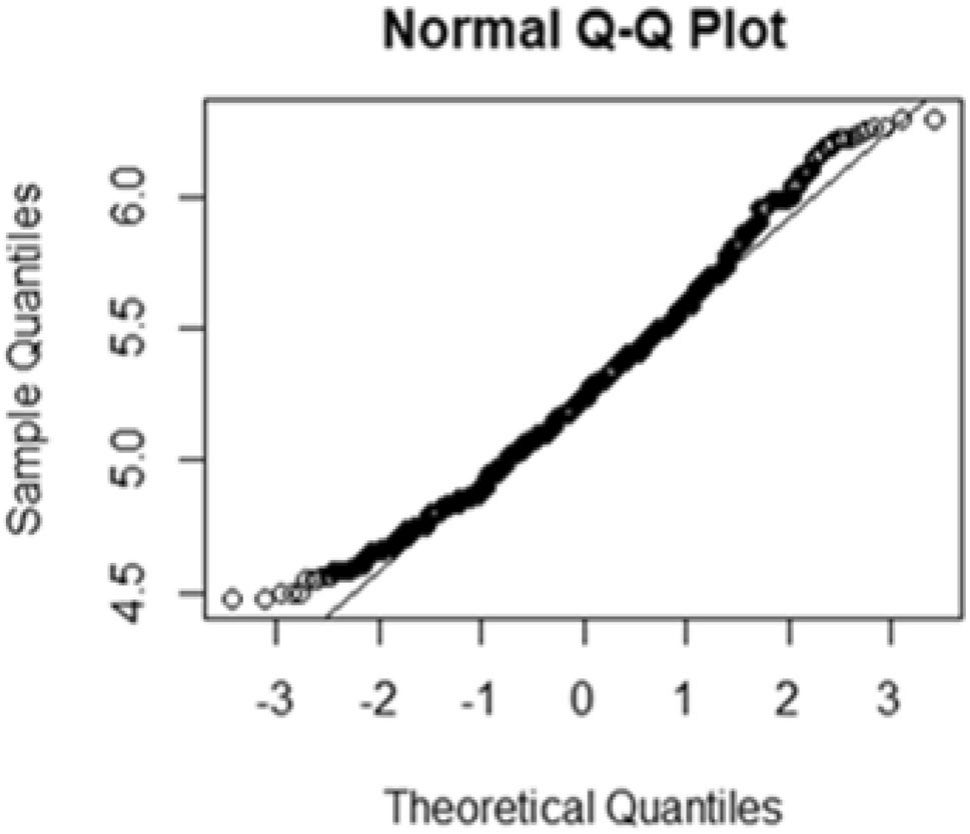
Q–Q plot of the lnFBS measurements over time.

Figures 2 and 3 displays the Q–Q plot for FBS measurement of the original data and natural logarithm transformed data respectively. The Q–Q plot for the original FBS showed that the normality assumption was violated and the FBS (Fasting Blood Sugar) was identified to exhibit the right-skewed shape of the distribution, suggesting that some transformation must be made to meet the assumptions. Therefore we used natural log transformation. The above-normal Q–Q plot for the logarithm of the actual data seems to approximately satisfy the assumption of normality or the logarithm transformed attained normality proximally. Thus, the analysis of this study used the natural logarithm transformed FBS data.

### Profile analysis

**Figure 4:**
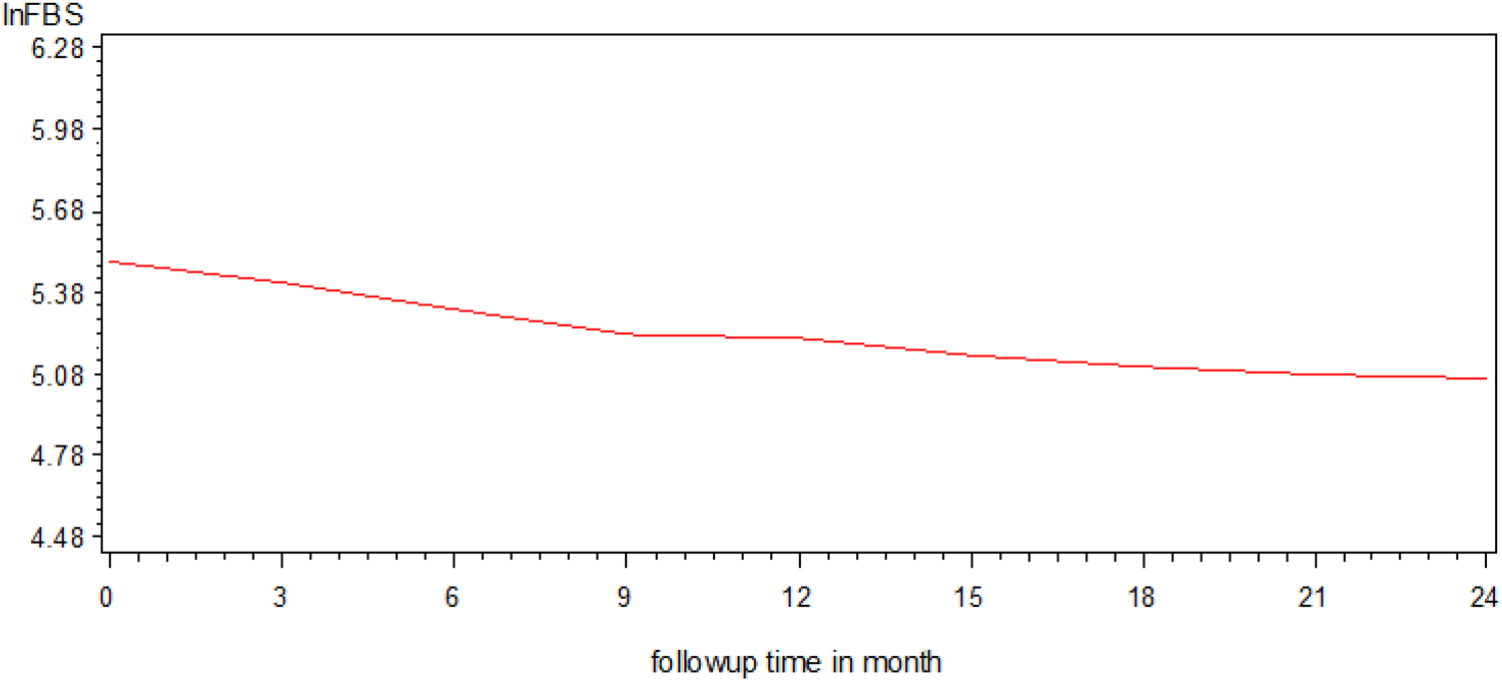
The overall mean profiles of T1DM patients over follow-up time.

Figure 4 displays the overall mean of lnFBS measurements of patient’s overtime. The plot demonstrates the variability within patients in lnFBS over time. As we have seen in the plot, the lnFBS of the patients at its initial time was high (mean lnFBS at 0 = 5.49 with 95% CI 5.46 to 5.53). After they start the treatment, the mean lnFBS decreased over time (e.g. mean lnFBS at 3 = 5.42 with 95% CI 5.38 to 5.46; mean lnFBS at 6 = 5.32 with 95% CI 5.28 to 5.36; mean lnFBS at 9 = 5.22 with 95% CI 5.18 to 5.27; mean lnFBS at 12 = 5.21 with 95% CI 5.17 to 5.26; mean lnFBS at 15 = 5.15 with 95% CI 5.10 to 5.20; mean lnFBS at 18 = 5.11 with 95% CI 5.06 to 5.16; mean lnFBS at 21 = 5.08 with 95% CI 5.02 to 5.14; mean lnFBS at 24 = 5.07 with 95% CI 4.99 to 5.15). This implies treatment had an impact on decreasing the FBS level of T1DM patients in DTGH. Also, the plot was decreased linearly in a straight-line fashion. This indicates that the linearity assumption of the transformed fasting blood sugar measurement was satisfied. Furthermore, the average values of fasting blood sugar decreased as the visit time (follow-up time) increased.

### Model building process

#### Selection of covariance structure in linear mixed model

To identify the appropriate covariance structure, the three commonly used covariance structures which are compound symmetry (CS), unstructured (UN), and first-order autoregressive AR (1) were considered. Among three covariance structure, UN structure was selected because of its smaller AIC and BIC value (e.g. AIC for UN = − 711.4, BIC for UN = − 625.7, AIC for CS = − 709.3, BIC for CS = − 618.2, AIC for AR (1) = − 707.4, BIC for AR (1) = − 647.1).

#### Selection of random effects in linear mixed model

Since in selection of covariance structure unstructured covariance structure was the best. So depending on it, we implemented different linear mixed models to study the longitudinal outcome by including the subject-specific random effects. Finally, we compared the information criteria values for the selection of random effects to be included in the linear mixed-effects model.

As we have seen from Table 2 result, we have chosen the random intercept and slope model to allow the intercept and coefficient to vary randomly among individuals. In another word, individual lnFBS of T1DM patients vary from visit to visit randomly. Therefore, the random intercept and random slope model was better to the data for the linear mixed-effects model on the basis of its lower values of AIC and BIC.

**Table 2:** Selection of random effects to be included in the linear mixed model.

Models for random effect	AIC	BIC	LogLik
Random intercept	− 711.3898	− 625.6995	371.6949
Random slope	− 904.6019	− 818.9117	468.301
Random intercept and slope	− 1082.224	− 985.8226	559.1121

### Model selection

In Table 3 the null model was the model fitted without covariate whereas the full model was the model fitted with all covariates considered for the model. Therefore, the full model was a better fit to the data based on a significant likelihood ratio test as well. And also the AIC and BIC values are smaller.

**Table 3:** Linear mixed model comparison.

Model	AIC	BIC	LogLik	LRT	P-value
Null model	− 573.3	− 556.6	291.7		
Full model	− 1082.2	− 1022.0	559.1	646.19	< 0.0001

### The linear mixed model analysis

For Fasting Blood Sugar level continuous measurement, a linear mixed model was used as is indicated in Table 4. As we have seen from Table 4 the variables family history of diabetes mellitus, age, hemoglobin, and visit time in months were significant at 5% level of significance for the final linear mixed model. Also, all the random effect parameters were statistically significant.

**Table 4:** Final linear mixed model result of longitudinal lnFBS.

Covariate	Estimate	SE	95% CI		P-value
			Lower	Upper	
Intercept	4.4313	0.1309	4.1733	4.6842	< 0.0001*
Age	0.0027	0.0012	0.0004	0.0051	0.0250*
**Gender(ref = female)**					
Male	0.0632	0.0329	− 0.0012	0.1261	0.0555
**Residence (rural)**					
Urban	− 0.0774	0.0415	− 0.1566	0.0037	0.0636
**Related disease (ref = no)**					
Yes	0.0613	0.0302	0.0019	0.1326	0.0440*
**Family history of DM (ref = no)**					
Yes	0.0634	0.0311	0.0024	0.1242	0.0431*
Hemoglobin	− 0.0625	0.0192	− 0.1002	− 0.0246	0.0013*
Creatinine	0.0498	0.0414	− 0.0313	0.1312	0.2301
Visit time	− 0.0255	0.0012	− 0.0276	− 0.0232	< 0.0001*

Random effects	SD	95% CI			
		Lower	Upper		
Intercept (${b}_{0i}$ )	0.1446	0.1256	0.1664		
Visit time (${b}_{1i}$ )	0.0159	0.0140	0.0181		
Corr $\left({b}_{0i},{b}_{1i}\right)$	− 0.5136	− 0.6369	− 0.3649		
Residual (${\varepsilon }_{i}$)	0.1337	0.1282	0.1394		

*SE* Standard error, *SD* Standard deviation; *Stands for statistically significant variable at 5% level of significant.

The estimate of the mean lnFBS level among T1DM patients in DTGH was 4.4313 mg/dl by keeping all other variables constant (P-value = 0.0001). Among T1DM patients in DTGH the mean lnFBS level declines by − 0.0255 mg/dl units per visit time of patients by keeping all other variables constant (P-value = 0.0001). The mean lnFBS level among T1DM patients in DTGH with comorbidity is 0.0613 mg/dl units higher than the mean lnFBS level among T1DM patients in DTGH without comorbidity by keeping all other variables constant (P-value = 0.0440). Among T1DM patients in DTGH, the mean lnFBS level increased by 0.0027 mg/dl units per age in the year of patients by keeping all other variables constant (P-value = 0.0250). The mean lnFBS level among T1DM patients in DTGH with a family history of diabetes is 0.0634 mg/dl units higher than the mean lnFBS level among T1DM patients in DTGH with no family history of diabetes by keeping all other variables constant (P-value = 0.0431). Among T1DM patients in DTGH the mean lnFBS level declines by − 0.0625 mg/dl mg/dl units per unit increase in hemoglobin level by keeping all other variables constant (P-value = 0.0013).

In this model there are three estimated variance components: var (${\varepsilon }_{i}$) = (0.1337)^2^ = 0.018, var(${b}_{0i}$) = (0.1446)^2^ = 0.021, and var(${b}_{1i}$ ) = (0.0159)^2^ = 0.0003. The total variation in fasting blood sugar level is estimated as 0.018 + 0.021 + 0.0003 = 0.0393 and the proportion of total variation that is attributed to; within-patient variability is $\frac{0.018}{0.0393}\times 100\%=46\%$, among patient variation in their mean fasting blood sugar level is $\frac{0.021}{0.0393}\times 100\%=53\%$, and among patient variation in their mean fasting blood sugar level due to random effect (visit time) is $\frac{0.0003}{0.0393}\times 100\%=1\%$ (Table 4).

## Discussion

The main objective of this study was to identify factors that affect the longitudinal fasting blood sugar of T1DM patients attending the OPD section at Debre Tabor General Hospital, Debre Tabor, Ethiopia using linear mixed model analysis. Age was an important clinical variable of lnFBS implies that the average lnFBS increases as age increases. This result was consistent with another study^19^. In their finding, old patients have poor glycaemic control as compared to younger patients. The results from these studies showed that among T1DM patients with a similar level of glycemia, iron deficiency anemia was associated with higher concentrations of HbA1c. However, this result was contradicted with another study^20^, that patients having low hemoglobin levels attained their optimal glycaemic control as compared to patients having high hemoglobin levels. The average lnFBS was found to evolve differently between patients with comorbidity and patients without comorbidity. The average lnFBS was higher for patients who had comorbidity compared to patients who had comorbidity. That means, patients, having other comorbidities poorly controlled their blood sugar as compared to patients with no comorbidity. This result was consistent with other studies^10^, for patients with comorbid cases attaining optimal glycaemic goals was difficult. Family history of diabetes mellitus was an important variable for lnFBS. Average lnFBS was higher for patients whose families had diabetes mellitus compared to patients whose families had no history of diabetes mellitus. This supported the finding of^7^ that patients who had no family history of diabetes can monitor their sugar levels well. In this study, the mean lnFBS of the patients at its initial time was high. After they start the treatment, the lnFBS decreased over time. This result was consistent with other studies^10,11,19^, in their finding as time increase, patients who follow their treatment properly could monitor their sugar and live longer. However, this result was contradicted with another study^21^, in their finding as time increase, the patients reached hyperglycemia and difficult to control their blood sugar.

### Strengths and limitations of this study

The study provides information on the blood sugar level of T1DM patients in DTGH by analyzing the data using an advanced methodology. It also set a solution for diabetes mellitus patients in DTGH, and it was used as an input for other studies. This study also provides evidence-based recommendations for revising and formulating public health policy and practice in Ethiopia. Furthermore, the study demonstrates the variability within and between T1DM patients in FBS over time. All general hospitals in Ethiopia may not provide the same patient information charts or medical records. In addition, patients may have not similar characteristics or backgrounds. Therefore, our study findings may not infer to other general hospital patients in Ethiopia. In DTGH, there may be poor patient’s medical record system which may lead biased inference.

## Conclusion

This retrospective study indicated that the overall variation in fasting blood sugar level among T1DM patients in DTGH was attributed to changing the characteristics of patients both in fixed effect and random effects of the model. Major contributors to the increment of fasting blood sugar level were increasing age, decreasing hemoglobin, having comorbidity, and belonging to a family with a diabetes history. The overall within and between variability in fasting blood sugar levels among T1DM patients in DTGH were high. Intervention measures at the DTGH level should be undertaken using health education and other measures by providing an emphasis on the prevention, early detection, and treatment of diabetes mellitus. The stakeholders should make this chronic illness part of their health agenda, and they should plan timely interventions. We recommend for health professionals in DTGH to include and record other determinant factors such as body mass index, feeding style, physical activity, etc.… for fasting blood sugar levels of T1DM patients. Researchers should do other studies on the determinant of fasting blood sugar levels of T1DM patients by considering such variables and other environmental factors (altitude and temperature) that were not included in this study.

## Data Availability

The data used for the current investigation is available in the hands of the corresponding author and will be submitted upon request.

## Abbreviations

T1DM: Type I diabetes mellitus
DTGH: Debre Tabor General Hospital
OPD: Outpatient Department
T2DM: Type II diabetes mellitus
AIDS: Acquired immune deficiency syndrome
WHO: World Health Organization
IDF: International diabetes federation
DM: Diabetes mellitus
FBS: Fasting blood sugar
CS: Compound symmetry)
AR (1): First-order autoregressive
UN: Unstructured
LRT: Likelihood ratio test
AIC: Akaike's information criterion
BIC: Bayesian information criterion
lnFBS: Natural logarithm of fasting blood sugar measure
lmm: Linear mixed model
LogLik: Log-likelihood
